# Effects of serum irisin, neuregulin 4, and weight management on obese adolescent girls with polycystic ovary syndrome

**DOI:** 10.1042/BSR20211658

**Published:** 2021-09-03

**Authors:** Shunshun Cao, Yangyang Hu

**Affiliations:** 1 Pediatric Endocrinology, Genetics and Metabolism, The Second Affiliated Hospital and Yuying Children's Hospital of Wenzhou Medical University, Wenzhou, 325000 Zhejiang, China; 2 Reproductive Medical Center, Department of Obstetrics and Gynecology, The Second Affiliated Hospital and Yuying Children’s Hospital of Wenzhou Medical University, Wenzhou, 325000 Zhejiang, China

**Keywords:** neuregulin4, obese adolescent, polycystic ovary syndrome, serum irisin, weight management

## Abstract

The study is aimed at investigating the association of serum irisin, neuregulin 4 (NRG4), and anti-müllerian hormone (AMH) with adolescent obesity with polycystic ovary syndrome (PCOS) and the efficacy of weight management interventions. Serum levels of irisin, NRG4, AMH, sex steroid hormone, body mass index (BMI), serum insulin, and C-peptide were measured in 52 obese adolescent girls with PCOS (PCOS group) and 43 obese adolescent girls without PCOS (non-PCOS group). The levels of AMH, NRG4, serum irisin, sex steroid hormones, BMI, serum insulin, and C-peptide were evaluated in obese PCOS girls before and after one year weight management. The levels of AMH, serum insulin, NRG4, and total testosterone of PCOS group were significantly higher than those of non-PCOS group. On the contrary, serum irisin and serum C-peptide in PCOS group were significantly lower than that in non-PCOS group. The levels of fat mass, percent body fat, total testosterone, AMH, NRG4, and serum insulin in the obese girls with PCOS showed significant decreases compared with before weight management intervention. On the contrary, after one year of body weight management intervention, serum irisin and serum C-peptide was significantly increased. Adolescent obesity complicated with PCOS is significantly associated with glucose and lipid metabolism and sex steroid hormone disorders, but the exact pathophysiological and clinical features are highly variable. Weight management intervention can significantly improve the clinical symptoms and hematological indicators, serum irisin and NRG4 can be used as two essential biomarkers for evaluating weight management.

## Introduction

Irisin was first reported in a paper published in Nature in January 2012, which brought new hope to the field of obesity clinical treatment, as researchers discovered new hormones in another class of brown fat and trans fat types. Irisin is a myokine released mainly from skeletal muscle after exercise and is considered the main target for inducing adipose tissue transformation, thus increasing heat generation and energy consumption. Since its discovery, some scholars have reported [1] that irisin is associated with metabolic diseases, including obesity, type 2 diabetes mellitus (T2DM), PCOS. Most studies have shown that irisin is positively correlated with obesity index [2]. From the perspective of pathophysiology, irisin is still an attractive target for the treatment of metabolic diseases, although a large number of studies are still needed. Irisin is elevated in type 2 diabetes and is associated with elevated levels of E-selectin; therefore, irisin may constitute a potential new therapeutic opportunity in the areas of obesity and diabetes [3]. Similarly, NRG4, a protein molecule containing a region similar to epidermal growth factor, is expressed and secreted primarily in brown fat cells. By specific binding to EGF receptor ERBB-4, it plays an important role in diseases related to glucose and lipid metabolism and plays physiological functions such as stimulating cell proliferation, inhibiting cell apoptosis, and improving cell energy metabolism.

AMH is a glycoprotein that is a member of the transforming growth factor-β family and reflects the number of antral follicles. The expression of AMH is different in different stages of female growth and development, and its biological effects are also different. The most important clinical application based on its pathophysiological changes is to evaluate female ovarian reserve and provide a basis for the evaluation of female fertility. Compared with other traditional biological indicators, AMH has many obvious advantages in evaluating ovarian reserve and is the most accurate biomarker of ovarian aging. AMH reflects the trend of declining ovarian reserve with age earlier than FSH, E2, statin B, and antral follicle counting (AFC). Rotterdam diagnostic criteria include polycystic ovary morphology (PCOM) under ultrasound, but a growing number of reports suggest serum AMH as an alternative diagnosis for PCOS [4]. AMH were found to distinguish between normal ovaries, PCOM and PCOS, and higher serum AMH levels were found in patients with PCOS, reflecting the number of antral follicles and the inherent defects of individual granulosa cells [5].

According to international evidence-based guidelines, weight management is the first line of treatment for PCOS [6]. However, the factors associated with the amount of weight management in women with PCOS and the success of weight management interventions are not well understood [7]. It is reported that 1% of adolescents have PCOS, of which nearly 40–70% are overweight or obese [8]. Obese adolescents with PCOS had more severe hyperandrogenism and insulin resistance than normal-weight PCOS adolescents. The study observed that AMH increases in adolescent girls with PCOS and returns to normal with weight management, and AMH is associated with hyperandrogenemia [9].

First of all, in the physiological age of girls, often menstrual disorders. Second, the multifollicular ovary is a feature of normal puberty and is morphologically indistinguishable from the polycystic ovaries. Third, there are practical and ethical problems with transvaginal ovarian ultrasound in adolescents. Therefore, the diagnosis of polycystic ovary syndrome is a challenge for adolescents. According to the current literature report [10], it is suggested that AMH should be used as a biochemical marker for the diagnosis of adolescent PCOS.

At present, there are many studies on the correlation between AMH and adolescent PCOS. However, there have been few reports on serum levels of anti-mullerian hormone, irisin, NRG4, and interventions for weight loss in obese adolescent girls with polycystic ovary syndrome at home and abroad. The purpose of the present study was to determine the levels of serum irisin, NRG4, and AMH in adolescents with obesity complicated with PCOS, compare these levels, and understand their correlation with other metabolic and hormonal parameters, as well as the influence of weight management on them.

## Materials and methods

### Study population

Data from 52 obese adolescent girls with a diagnosis of PCOS (PCOS group) were compared with 43 obese adolescent girls without PCOS (non-PCOS group), All the cases were from the Department of Pediatric Endocrinology, Genetics and Metabolism and the Reproductive Medical Center of the Second Affiliated Hospital of Wenzhou Medical University, from 2015 to 2020. The present study was a prospective study and approved by the ethics committee of the Second Affiliated Hospital of Wenzhou Medical University. The patients in the study signed the informed consent form by their guardians and themselves. According to the latest international evidence-based guidelines [6], the diagnosis of PCOS is based on the presence of clinical symptoms of hyperandrogenism or biochemical hyperandrogenism, irregular menstrual cycles and ovulatory dysfunction excluding other causes [11]. Inclusion criteria: girls diagnosed with PCOS, age 12–18 years, and postmenarche BMI 95th percentile for the same age and sex. Those who were diagnosed with inherited metabolic disorders took drugs that affected glucose and lipid metabolism and did not accept strict weight management were excluded.

### Study schedule

All patients underwent a medical history, physical examination, height and weight, and hematologic and biochemical tests. The Tanner Stage was used to assess adolescent development, according to the study reported by Chulani et al. [12]. Serum AMH, irisin, NRG4, sex steroid hormone, C-peptide, and insulin were detected in fasting blood samples of the two groups and PCOS group before and after weight management, respectively. Sex steroid hormone testing was done during the menstrual or follicular period. Serum anti-Mullerian hormone, NRG4, and irisin were tested in the laboratory of the Center for Reproductive Medicine, and the remaining blood indicators were tested in the Center for Blood Biochemistry. BMI was calculated based on the measured height and weight. The formula of body fat [body fat% = 0.546× (subscapularis skinfold thickness + triceps skinfold thickness in mm) + 9.7] was calculated according to the method reported by Reinehr et al. [9]. The PCOS group will undergo a one-year multi-component lifestyle intervention for weight management that includes diet control, increased exercise, and reduced sedentary behavior.

### Methodology of serum irisin detection

Serum irisin was measured by double antibody one-step sandwich enzyme-linked immunosorbent assay (Human Irisin Kit Xlpcc® ELISA, Xinle Biology, Shanghai). The samples, standard samples, and HRP-labeled detection antibodies were added into the coated micropores precoated with irisin capturing antibodies, and the samples were incubated and thoroughly washed. The substrate TMB, catalyzed by peroxidase, converted to blue and converted to final yellow under the action of acid. The intensity of the color was positively correlated with irisin in the sample. The absorbance (OD value) was measured at 450 nm with a microplate analyzer, and the concentration of the sample was calculated.

### Methodology of serum NRG4 detection

NRG4 was detected by double antibody sandwich assay (Human NRG 4 ELISA Kit, Wuhan Fine Biotech Co. Ltd). NRG4 antibody is coated on the plate. During the experiment, the NRG4 in the sample or standard is bound to the coated antibody, and the free component is washed away. Biotinylated anti-NRG4 antibodies and horseradish peroxidase labeled avidins were successively added. The anti-NRG4 antibody binds to the NRG4 binding to the coated antibody, and the biotin specifically binds to avidin to form an immune complex, and the free component is washed away. Add color substrate (TMB), TMB in the horseradish peroxidase catalyzed blue, after adding the stop solution turned yellow. OD value was measured at 450 nm with a microplate analyzer, and the concentration of NRG4 was directly proportional to the OD450 value. The concentration of NRG4 in the sample was calculated by drawing a standard curve.

### Methodology of AMH detection

Serum AMH was measured by chemiluminescence immunosandwich assay (Human AMH assay kit, Shenzhen snibe diagnostic Biomedical Engineering Co. Ltd). Automatic chemiluminescence analyzer (MAGLUMI 800) was purchased from Shenzhen snibe diagnostic Biomedical Engineering Co. Ltd. Streptomavidin was coated on magnetic microspheres, biotin labeled one anti-AMH monoclonal antibody (capturing antibody), and ABEI (illuminating agent) labeled with another anti-AMH monoclonal antibody (detecting antibody). Incubation: The sample were incubated with biotin markers, luminescent markers, and magnetic microspheres. Streptomavidin coated on magnetic microspheres binds to biotin labeled on anti-AMH monoclonal antibodies. In the samples, AMH was combined with biotin labeled anti-AMH monoclonal antibody and ABEI labeled anti-AMH monoclonal antibody, respectively, to form the ‘magnetic microspheres-streptoavidin-biotin-anti- AMH monoclonal antibody-AMH- anti-AMH monoclonal antibody-ABEI’ immune complex. After incubation, the unbound material is removed by magnetic separation cleaning. Finally, the substrate solution used in the automatic immune test system is added to initiate the chemiluminescence reaction and generate an optical signal. The relative light intensity measured by the photomultiplier tube has a positive correlation with the concentration of AMH in the sample.

### Statistical analyses

The descriptive characteristics of PCOS group and non-PCOS group were analyzed by Mann–Whitney *U* test based on homogeneity of variance, and the chi-square test was used for comparison of rates. Statistical analysis was performed using SPSS, (version 24.0, SPSS Inc, Chicago, U.S.A.). Data were expressed as mean±standard deviation (SD), and *P*-value < 0.05 was considered statistically significant.

## Results

Our study found that the age and Tanner stage of PCOS group (PCOS group age: 14.15 ± 0.31 vs. non-PCOS group age: 14.20 ± 0.15), (PCOS group tanner stage:0 (0%)/3 (6%)/49 (94%) vs. non-PCOS group tanner stage: 0 (0%)/2 (5%)/41 (95%)) were similar to those of non-PCOS group, and there was no statistical difference (*P*>0.05). However, the BMI (PCOS group: 37.22 ± 1.53 vs. non-PCOS group: 32.35 ± 1.24), fat mass (PCOS group: 46.17 ± 2.33 vs. non-PCOS group: 37.7 ± 2.15), percent body fat (PCOS group: 45.50 ± 0.62 vs. non-PCOS group: 39.33 ± 0.71), AMH (PCOS group: 5.46 ± 0.35 vs. non-PCOS group: 2.13 ± 0.27), serum insulin (PCOS group: 29.21 ± 13.63 vs. non-PCOS group: 18.28 ± 10.34), serum NRG4 (PCOS group: 8.12 ± 3.03 vs. non-PCOS group: 4.22 ± 1.25), total testosterone (PCOS group: 1.16 ± 0.28 vs. non-PCOS group: 0.76 ± 0.12) and serum C-peptide (PCOS group: 11.60 ± 3.14 vs. non-PCOS group: 4.23 ± 2.25) of PCOS group were significantly higher than those of non-PCOS group, and the difference was statistically significant (*P*<0.05). On the contrary, serum irisin (PCOS group: 256 ± 22.40 vs. non-PCOS group: 375 ± 18.65) in PCOS group was significantly lower than that in non-PCOS group, and the difference was statistically significant (*P*<0.05) (Table 1).

**Table 1:** Physical characteristics and blood parameters in obese PCOS group versus obese non-PCOS group

Variables	PCOS group, *n*=52	non-PCOS group, *n*=43	*P-*value
Age (years)	14.15 ± 0.31	14.20 ± 0.15	NS
BMI (kg/m^2^)	37.22 ± 1.53	32.35 ± 1.24	0.037
Tanner stage (III/IV/V),%	0(0%)/3(6%)/49(94%)	0(0%)/2(5%)/41(95%)	NS
Fat mass (kg)	46.17 ± 2.33	37.7 ± 2.15	0.028
Percent body fat (%)	45.50 ± 0.62	39.33 ± 0.71	0.041
FSH (IU/L)	5.91 ± 0.12	6.28 ± 0.78	0.262
LH (IU/L)	6.30 ± 0.64	5.67 ± 0.59	0.412
E2 (pg/ml)	56.10 ± 15.23	51.5 ± 18.54	0.347
Total testosterone (ng/ml)	1.16 ± 0.28	0.76 ± 0.12	0.017
AMH (ng/ml)	5.46 ± 0.35	2.13 ± 0.27	0.027
Serum irisin (ng/ml)	256 ± 22.40	375 ± 18.65	0.023
Serum NRG4 (ng/ml)	8.12 ± 3.03	4.22 ± 1.25	0.031
Serum C-peptide (ng/ml)	11.60 ± 3.14	4.23 ± 2.25	0.012
Serum insulin (uIU/ml)	29.21 ± 13.63	18.28 ± 10.34	0.026

Statistically significant *P*<0.05; Data are mean ± standard deviation.

Abbreviations: BMI, body mass index; E2, estradiol; FSH, follicle-stimulating hormone; LH, luteinizing hormone.

After data analysis, we found that after one year of body weight management intervention, fat mass (before group: 46.17 ± 2.33 vs. after group: 40.25 ± 3.14), percent body fat (before group: 45.50 ± 0.62 vs. after group: 40.14 ± 2.87), total testosterone (before group: 1.16 ± 0.28 vs. after group: 0.81 ± 0.17), AMH (before group: 5.46 ± 0.35 vs. after group: 2.28 ± 0.34), serum insulin (before group: 29.21 ± 13.63 vs. after group: 17.87 ± 9.37), serum NRG4 (before group: 8.12 ± 3.03 vs. after group: 4.83 ± 1.75), and serum C-peptide (before group: 11.60 ± 3.14 vs. after group: 6.45 ± 1.80) in PCOS group showed significant decreases compared with before weight management intervention, showing a significant difference (*P*<0.05). On the contrary, after one year of body weight management intervention, serum irisin (before group: 256 ± 22.40 vs. After group: 325 ± 31.74) was significantly increased, and the difference was statistically significant (*P*<0.05). Although obesity in PCOS girls improved significantly after first-line treatment with weight management, FSH (before group: 5.91±0.12 vs. after group: 5.39 ± 2.60), LH (before group: 6.30 ± 0.64 vs. after group: 7.26 ± 3.40), and E2 (before group: 56.10 ± 15.23 vs. after group: 61.17 ± 2.78) were similar to those before weight intervention without statistically significant differences (*P*>0.05) (Table 2).

**Table 2:** Physical characteristics and blood parameters of the PCOS group before and after weight management intervention

Variables (*N*=52)	Before	After	*P-*value
Fat mass (kg)	46.17 ± 2.33	40.25 ± 3.14	0.034
Percent body fat (%)	45.50 ± 0.62	40.14 ± 2.87	0.039
FSH (IU/l)	5.91 ± 0.12	5.39 ± 2.60	0.162
LH (IU/l)	6.30 ± 0.64	7.26 ± 3.40	0.221
E2 (pg/ml)	56.10 ± 15.23	61.17 ± 2.78	0.357
Total testosterone (ng/ml)	1.16 ± 0.28	0.81 ± 0.17	0.034
AMH (ng/ml)	5.46 ± 0.35	2.28 ± 0.34	0.023
Serum irisin (ng/ml)	256 ± 22.40	325 ± 31.74	0.028
Serum NRG4 (ng/ml)	8.12 ± 3.03	4.83 ± 1.75	0.041
Serum C-peptide (ng/ml)	11.60 ± 3.14	6.45 ± 1.80	0.036
Serum insulin (uIU/ml)	29.21 ± 13.63	17.87 ± 9.37	0.029

Statistically significant *P*<0.05; Data are mean ± standard deviation.

Abbreviations: E2, estradiol; FSH, follicle-stimulating hormone; LH, luteinizing hormone.

## Discussion

A cross-sectional study [13] by Bacopoulou et al. showed that serum irisin was significantly increased in lean adolescent patients with PCOS, and serum irisin was associated with metabolic and reproductive characteristics as well as high androgen phenotypes in adolescents. The results of the study were similar to our conclusions. We found that the total testosterone, serum insulin serum NRG4, and serum C-peptide of obese girls with PCOS were significantly higher than those of obese girls without PCOS. On the contrary, serum irisin in obese girls with PCOS was significantly lower than that in obese girls without PCOS. The authors of Bostanc et al. [14] also expressed a similar view, they found that the serum irisin and NRG4 level of PCOS patients were significantly higher than that of the control group of people, which may be related to insulin resistance, leptin resistance and metabolic syndrome. By analyzing the data, Wang et al. found that the serum irisin level of PCOS patients was significantly lower than that of non-PCOS patients, the serum irisin level of obese PCOS patients was significantly lower than that of non-obese PCOS patients; however, the serum irisin level of PCOS patients with dyslipidemia was significantly higher than that of PCOS patients with normal lipids [15].

Tokmak et al. showed that the serum AMH level of non-obese adolescent females with PCOS and insulin resistance was higher than that of PCOS patients without insulin resistance, and there was a significant positive correlation between the serum AMH level and insulin resistance, which was similar to the results of our study [16]. Our study showed that the serum AMH levels and fasting serum insulin levels in obese girls with PCOS were significantly higher than those in obese girls without PCOS. Studies have shown that the level of AMH in obese girls with PCOS is almost twice as high as that in obese girls without PCOS [10]. However, it has been reported in other literature that there is no difference in serum AMH between obese and non-obese adolescents with PCOS [17]. A prospective study [18] by Skarba et al. revealed that serum AMH levels are associated with insulin resistance but not BMI, and the increase in AMH reflects the disorder of gonadotropin release in patients with PCOS. Our study found that the serum total testosterone levels in obese girls with PCOS were significantly higher than those in obese girls without PCOS. We suggest that the elevated serum total testosterone level in obese girls with PCOS is related to the disturbance of gonadotropin.

In a cross-sectional study [19], Kurek Eken et al. showed that serum NRG4 level was independently related to BMI through multiple regression analysis. Obesity was the biggest factor affecting NRG4 secretion in PCOS patients, and weight management was the key factor to solve metabolic abnormalities and fertility problems related to PCOS. Obesity and PCOS share many of the same metabolic disorders, such as hyperandrogenism and hyperinsulinemia with insulin resistance, according to a meta-analysis study that highlights the importance of obesity prevention in adolescent PCOS treatment through weight intervention and in combination with lifestyle changes [20]. This is similar to the results of our study. Through data analysis, the serum total testosterone levels and serum insulin in obese girls with PCOS were significantly higher than those in obese girls without PCOS, and weight management could significantly improve it. Based on our study, we found that a small number of subjects did not adhere strictly to weight management guidelines. Therefore, we excluded this part of the study. The few subjects who did not adhere to strict weight management did not achieve the expected results in the test parameters. Therefore, strict weight management is essential to improve the obese adolescent girls with PCOS. The number of subjects who did not adhere to strict weight management was too small to be statistically compared with those who did.

Serum C-peptide is a substance produced synchronously in the process of insulin secretion by the human body, and the amount of it produced is in a 1:1 relationship with the newly produced insulin. C-peptide detection is often used in the classification and diagnosis of diabetes, and C-peptide can truly reflect the actual insulin level, so the body produces as much insulin as it synthesizes. Our data showed that serum C-peptide and serum insulin levels were positively correlated, and the obese girls with PCOS group were significantly higher than the obese girls without PCOS. After weight management intervention, serum C-peptide and serum insulin levels in the obese girls with PCOS group were significantly reduced. Through data analysis, Ke et al. found that hyperinsulinemia in patients with PCOS was caused by increased pancreatic β-cell secretion and impaired liver clearance of these hormones [21].

Our study found that obese adolescent girls with PCOS often showed obvious characteristics of high androgen in sex hormone disorders. In terms of metabolic disorders, in addition to the body surface features of overweight and increased body fat, hematological indicators showed that serum insulin, serum NRG4, and serum C-peptide were significantly increased, and serum irisin was significantly decreased. Weight management can improve sex hormone and metabolic disorders. The data indicate that these parameters may be used as clinical indicators for the diagnosis of obesity with PCOS and the evaluation of treatment effect, and these results have clear clinical application value.

## Conclusion

Although the definition and diagnostic criteria of adolescent PCOS are still controversial, AMH remains a essential diagnostic biomarker. Adolescent obesity with PCOS is associated with glucose and lipid metabolism and sex steroid hormone disorders. Serum irisin can be used as an important indicator of lipid metabolism, but its exact pathophysiological and clinical characteristics vary greatly. Weight management intervention can significantly improve the clinical symptoms and hematological indicators, serum irisin can be used as a essential biomarker to evaluate weight management. However, our study was limited by the low prevalence of such diseases and patient compliance. This is a single-center study. The number of enrolled patients is not large enough, and further multicenter studies are needed to verify it.

## Data Availability

The data that support the findings of this study are available from the corresponding author upon reasonable request.

## Abbreviations

AFC: antral follicle counting
AMH: anti-müllerian hormone
BMI: body mass index
FSH: follicle-stimulating hormone
LH: luteinizing hormone
NRG4: neuregulin 4
PCOM: polycystic ovary morphology
PCOS: polycystic ovary syndrome
T2DM: type 2 diabetes mellitus
